# An Investigation on the Thermal Effusivity of Nanofluids Containing Al_2_O_3_ and CuO Nanoparticles

**DOI:** 10.3390/ijms130810350

**Published:** 2012-08-20

**Authors:** Monir Noroozi, Azmi Zakaria, Mohd Maarof Moksin, Zaidan Abd Wahab

**Affiliations:** Department of Physics, University Putra Malaysia, UPM Serdang 43400, Selangor D.E., Malaysia; E-Mails: monir.noroozi@gmail.com (M.N.); maarof@science.upm.edu.my (M.M.M.); zaidan@science.upm.edu.my (Z.A.W.)

**Keywords:** pyroelectric, thermal diffusivity, nanoparticles, thermal properties

## Abstract

The thermal effusivity of Al_2_O_3_ and CuO nanofluids in different base fluids, *i.e.*, deionized water, ethylene glycol and olive oil were investigated. The nanofluids, nanoparticles dispersed in base fluids; were prepared by mixing Al_2_O_3_, CuO nanopowder and the base fluids using sonication with high-powered pulses to ensure a good uniform dispersion of nanoparticles in the base fluids. The morphology of the particles in the base fluids was investigated by transmission electron microscopy (TEM). In this study, a phase frequency scan of the front pyroelectric configuration technique, with a thermally thick PVDF pyroelectric sensor and sample, was used to measure the thermal effusivity of the prepared nanofluids. The experimental results of the thermal effusivity of the studied solvents (deionized water, ethylene glycol and olive oil) showed good agreement with literature values, and were reduced in the presence of nanoparticles. The thermal effusivity of the nanofluid was found to be particularly sensitive to its base fluid and the type of nanoparticles.

## 1. Introduction

A large amount of research is focused on metal oxide nanoparticles (NPs) which are widely used in various and a broad range of industrial applications. Alumina (Al_2_O_3_) is considered an essential substrate in electronic device fabrication [1] and CuO NPs are used in gas sensors, catalysts, superconductors, and ceramic pigments [2] Metal oxide NPs dispersion in liquids is of fundament and technological importance for nanofluid applications such as antibacterial medical treatment and thermal management systems because of its transport property enhancement. The thermal properties of nanofluids have been the subject of an intensive study in nanotechnology applications [3]. The two thermal properties, thermal diffusivity *α* and effusivity *e* are connected with other thermal properties, thermal conductivity *k* and volumetric specific heat *c_p_* by *α = k*/*ρc_p_* and $e=k/\sqrt{\alpha }$ where *ρ* is the density of the sample [4]. The thermal effusivity essentially measures the thermal impedance of the selected sample for the heat transfer in the environment [5]. In the pyroelectric (PE) technique, the temperature variation of a sample, exposed to modulated radiation, is measured with a PE sensor [6]. There are practically two detection configurations proposed for the PE technique; the back pyroelectric configuration (BPE) when a modulated light impinges on the surface of the sample, and the front pyroelectric configuration (FPE) when light impinges on the surface of a PE sensor [7]. All four thermal parameters (specific heat, thermal diffusivity, conductivity and effusivity) can be measured by combining all the different particular cases [8]. Concerning the front configuration, until now, two detection regimes were proposed; the thermally thin film sensor (PVDF) [9] and the thermally thick ceramic sensor (LiTaO_3_) [10] regimes. In both cases, the sample must be thermally thick. In principle, the thermal parameters of the sample are obtained from the analysis of the phase or amplitude of the complex signal generated when the sample is periodically heated in a frequency scan. However, the phase of the signal leads to more accurate results than that of the amplitude [10]. The FPE configuration, for the case of the thermally thick sensor scheme, the thermal effusivity of liquid mixtures, liquid foodstuffs and magnetic nanofluids have been measured [5,7–12].

Normally, most studies of nanofluids are focussed on thermal conductivity and its relationship with other relevant parameters such as type of NPs, carrier fluid, surfactant, concentration and NPs size, *etc*. [13–19]. The suitability of observing the changes of thermal parameters with regard to these nanofluids parameters is due to their unique optical and thermal properties that are not yet completely understood. Here, we report a study on the thermal effusivity and structure of two metal oxide NPs, Al_2_O_3_ and CuO, dispersed in three different base fluids, deionized water (DW), ethylene glycol (EG) and olive oil. The FPE configuration was chosen to measure this thermal parameter by using a frequency scan of the phase of the signal employing PVDF as the PE sensor in a thermally thick regime.

## 2. Results and Discussion

### 2.1. Properties of Commercial Nanoparticles

**Figure 1:**
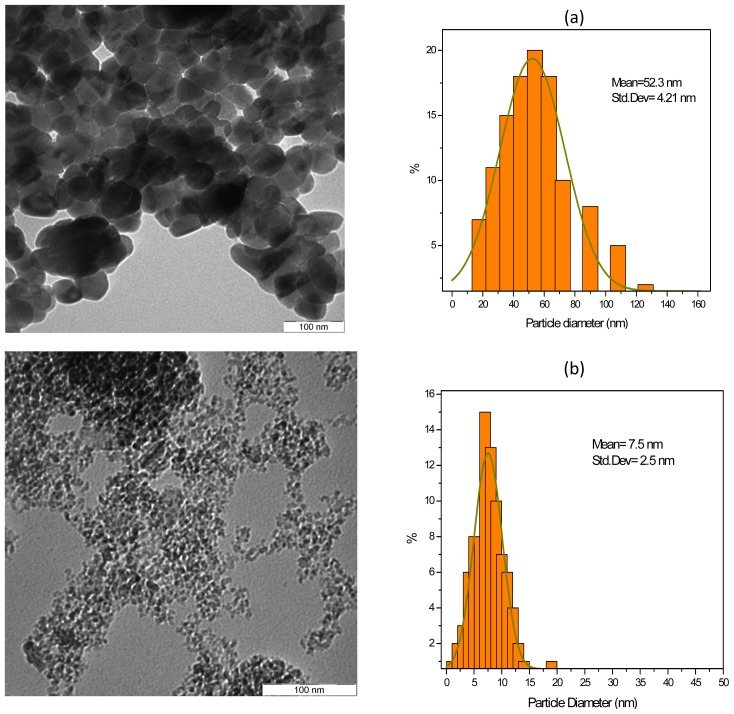
Transmission electron microscopy (TEM) image of (**a**) CuO particles with a nominal size of 50 nm, the mean diameter of these particles is 52 nm and (**b**) alumina particles with a nominal size of 11 nm, the mean diameter of these particles is 7.5 nm as determined by TEM.

Figure 1 shows TEM images and their corresponding size distributions of CuO and Al_2_O_3_ NPs in water. The mean diameter and the size distributions of NPs obtained by measuring the diameter and standard deviation of particles in the images are shown beside each image. CuO and Al_2_O_3_ nanofluids showed excellent stability with 30 min of sonication as can be seen, remaining stable with only minor settling after a week at room temperature. However, according to TEM images, NPs were not well dispersed and some agglomerates were present (Figure 1) as the image measurement requires a dry sample under high vacuum conditions. The mean particle sizes of CuO and Al_2_O_3_ NPs observed in TEM images are about 7.5 ± 2.5 and 52.3 ± 4.2 nm, respectively; indicating that these commercial metal oxide particles are in the nanoscale range. However, the particle sizes of these commercial NPs determined from TEM images are slightly different to those reported by the manufacturers. Particles in each sample were aggregated to some degree. While CuO particles have a spherical shape with some uniform size distribution as reported by the producer, the alumina particles are irregularly shaped with considerable polydispersity and probably porous morphology.

### 2.2. Thermal Effusivity Measurements

The symbols represent the experimental data and the solid lines are theoretical fits to the normalized phase of the PE signal [20]. Given as:

(1) $$\theta =\text{arctan}\left[\frac{A{\text{e}}^{-L_{p}/\mu_{p}}\;\text{sin}\left(L_{p}/\mu_{p}\right)}{1-A{\text{e}}^{-L_{p}/\mu_{p}}\;\text{cos}(L_{p}/\mu_{p})}\right],\;\;\;A=1+R_{sp}$$

with $\mu_{i}=\sqrt{\frac{\alpha_{i}}{\pi f}}$, where *μ_i_*, *α_i_*, *l_i_* and *f* are the thermal diffusion length*,* the thermal diffusivity, the thickness of material *i*, and the chopping frequency, respectively. The geometrical configuration consists of the thermally thick sensor or sample (*L*_i_/*μ*_i_) ≫ 1, and the subscript *i* can be PE sensor (*p*) and liquid sample (*s*). *A* can be determined by fitting Equation 1 from the plot of phase signal *θ*(*f*) versus frequency *f*. Then from *A* = *1 + R_sp_* the thermal effusivity *e* can be determined from the reflection coefficient, *R_sp_*, of the thermal waves between two media (*s* and *p*),

(2) $$R_{sp}=\left(e_{s}-e_{p}\right)/\left(e_{s}+e_{p}\right)$$

**Figure 2:**
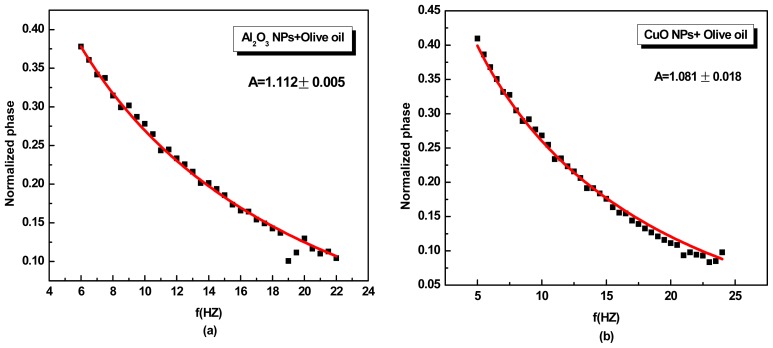
The pyroelectric (PE) normalized phase of Al_2_O_3_ + olive oil (**a**) and CuO + olive oil; (**b**) as a function of the light modulation frequency. Dots are experimental data and the solid line is the best fit to Equation 1.

**Figure 3:**
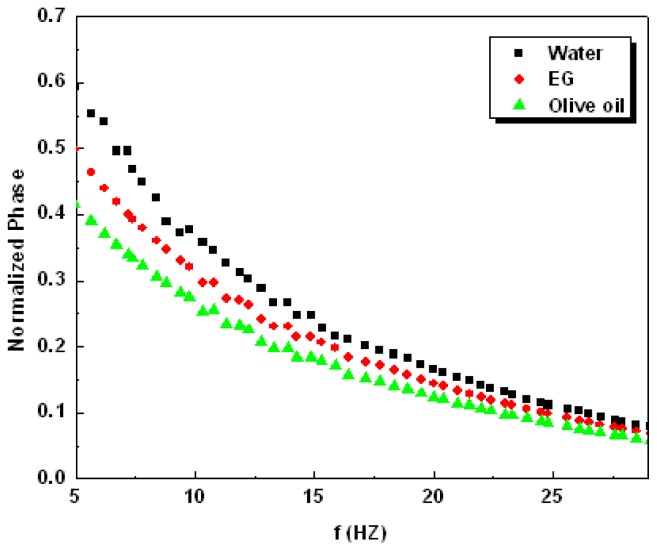
The frequency behaviour of the normalized phase of the PE signal of pure solvents such as water, ethylene glycol (EG) and olive oil.

Figure 2 shows the PE normalized phase of Al_2_O_3_ + olive oil (a) and CuO + olive oil (b) as a function of the light modulation frequency at a modulation frequency between 5 to 25 Hz. From this fit the parameter *A* = (1.112 ± 0.005) and (1.175 ± 0.006) was obtained, corresponding to thermal effusivity values of (0.614 ± 0.003) × 10^3^ Ws^1/2^m^−2^K^−1^ and (0.697 ± 0.003) × 10^3^ Ws^1/2^m^−2^K^−1^ for olive oil that contained Al_2_O_3_ and CuO NPs, respectively, obtained by using Equation 2. In Figure 3, the normalized phase curve magnitude of the PE signal depends on *e_s_*/*e_p_* ratio, where a very small value of normalized phase comes from olive oil due to its small ratio. This is followed by pure solvents EG and then water.

**Figure 4:**
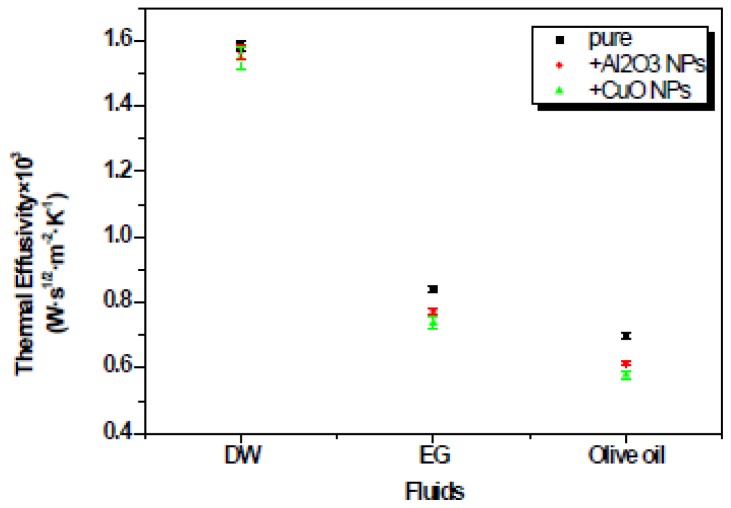
Comparison of the thermal effusivities and standard error bar of Al_2_O_3_ and CuO nanoparticles (NPs) in different base fluids (deionized water (DW), EG and olive oil) with the pure solvent.

The effusivity of all nanofluids and their comparison with pure solvents are also displayed in Table 1 and Figure 4.

### 2.3. Discussion

The thermal effusivity value obtained for water, as a standard sample, differed only by 0.5% with values described in the literature [13]. The thermal effusivity of EG and olive oil also are close to the literature values reported for pure solvents [21]. Therefore the FPE configuration can be considered reasonably reliable to measure this thermal parameter. As can be seen, the nanofluid thermal effusivity depends both on the base fluids (DW, EG and olive oil) and the NPs (Al_2_O_3_ and CuO), however the NPs influence is less than that of the base fluids. It is clear, that the accuracy of the thermal effusivity decreases with the increase in standard deviation of the fit performed with Equation (1). It is possible to observe that the thermal effusivity showed a behavior opposed to the results for the thermal diffusivity, which is due to the fact that both physical properties are inversely proportional ( $e=k/\sqrt{\alpha }$) [22].

The base fluid with a high thermal diffusion length, such as DW has a high frequency limit [23]. Experimentally, only the lower frequency limit is rather clear, established by theory, while the upper frequency limit depends on the experimental facilities as the signal-to-noise (S/N) ratio decreases. Consequently, thermal effusivity is more precise in the case of a low thermal diffusivity [21] for the base fluid and NPs for nanofluids, such as Al_2_O_3_ NPs dispersed in olive oil. We found about 1–2% relative error in the thermal effusivity of a nanofluid with DW with high thermal effusivity, and about 0.5–1.7% relative error in the thermal effusivity of the nanofluid with olive oil with a low thermal effusivity. The result shows that the relative error of the measurement is less than 2%. The main particularities of the method responsible for the high accuracy of the results are: that the information is contained in the phase of the PE signal and the values of the thermal parameters are the results of the fitting procedures.

## 3. Experimental Section

In order to prepare the nanofluid sample, 0.125.wt% of Al_2_O_3_ NPs (11 nm, 99%, Nanostructured and Amorphous Materials, Inc.) was dissolved in DW (or EG or olive oil) and stirred vigorously with magnetic stirring for 1.h until a clear solution was obtained. Then the solution was sonicated using a probe sonication (VCX.500, 20 kHz, 500 W) with high-powered pulses for about 30 min to ensure a good uniform dispersion of NPs in the base fluids. The same procedure was followed for the preparation of CuO NPs (50 nm, 99%, Nanostructured and Amorphous Materials, Inc.), which were dispersed in DW (or EG or olive oil). Average particle sizes provided by the manufacturer were typically determined by surface area measurements of the dry powder. However, different lots of particles may have different sizes, and this measurement provides no guaranteed information regarding the size distribution of the particles. Thus, transmission electron microscopy (TEM) was employed to obtain images of CuO and Al_2_O_3_ particles and to determine the average particle size. The individual particle size and distributions were determined using the UTHSCSA Image Tool software, version 3.0 (The University of Texas Health Science Center: San Antonio, TX, USA).

**Figure 5:**
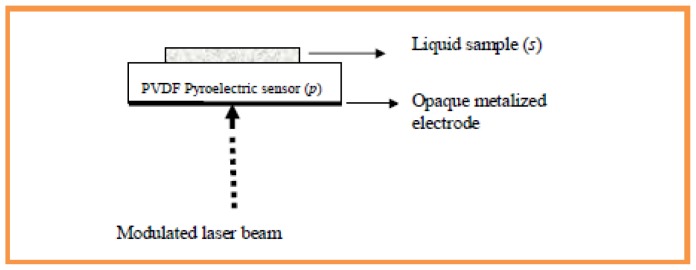
Schematic view of the front pyroelectric configuration (FPE) detection cell.

The FPE technique was used to measure thermal effusivity of the nanofluids. A schematic view of the FPE cell is presented in Figure 5.

The radiation source was a 30 mW He-Ne laser, modulated by a mechanical chopper (SR.540) and illuminating the bottom side of the PVDF PE sensor. A 52 μm thick metalized PE sensor (MSI DT1-028K/L) was coated with a thin layer of black paint of about 10 μm as an efficient light to heat converter. The PE voltage signal produced from the generated thermal wave was analyzed by a lock-in amplifier (SR.530) to produce both amplitude and phase signals. The detection cell in this configuration was actually composed of the PE sensor situated in a cavity that can be filled with 1.mm of fluid sample, which simply fills a plastic ring of 1.cm, glued onto the top side of the sensor. The filling and the discharge of the cavity, and the cleaning procedure, can be performed without removing the sensor, so the illuminated area is always the same. The frequency scan was performed in the 5–25 Hz frequency range, with a frequency step of 0.5 Hz, in which the sample was thermally thick. The PE phase signal was normalized to the one obtained with air instead of the sample, in order to eliminate parameters that are difficult to estimate experimentally. A PC was used for data acquisition and the S/N ratio of the measurement was better than 750. The measurements were performed shortly after sample preparation at room temperature. Due to the limited volume of the detection cell and the short measurement interval, the concentration of the solutions remained constant during the experiment. The advantages of this method are low cost of the detection cell, ease in implementation and requiring only a small quantity of liquid sample. Additionally, scanning is complete within several minutes, therefore sedimentation and/or aggregation, if any, can be avoided.

## 4. Conclusions

The proposed FPE configuration of using PVDF as a thermally thick sensor is reasonably accurate for the measurement of thermal effusivities by utilizing its phase signal. This configuration was later applied to measure the thermal effusivity of nanofluids that contained Al_2_O_3_ and CuO NPs in various base fluids. The thermal effusivity value is more precise in the case of a low thermal diffusivity of the base fluid. The thermal effusivity of different liquids, mixed with NPs decreases compared to those of the pure liquids.

## Figures and Tables

**Table 1:** Experimental thermal effusivity for solvents with Al_2_O_3_ and CuO nanoparticles (NPs) and thermal effusivity values of pure solvents from the literature. The standard error of thermal effusivity (Δ*e*) was calculated from Δ*e* = *e*(Δ*A*/*A*).

NPs	Base fluid	Fitting parameter(*A*)	Thermal effusivity × 10^3^ (Ws^1/2^m^−2^K^−1^) measurement	Relative error%	Thermal effusivity × 10^3^ (Ws^1/2^m^−2^K^−1^) Literature
Al_2_O_3_	water	1.523 ± 0.014	1.566 ± 0.015	0.95	-
Al_2_O_3_	EG	1.223 ± 0.009	0.773 ± 0.006	0.77	-
Al_2_O_3_	olive	1.112 ± 0.005	0.614 ± 0.003	0.48	-
CuO	water	1.519 ± 0.028	1.547 ± 0.029	1.87	-
CuO	EG	1.202 ± 0.021	0.738 ± 0.012	1.75	-
CuO	olive	1.081 ± 0.018	0.577 ± 0.009	1.56	-
-	water	1.528 ± 0.011	1.586 ± 0.011	0.69	1.579 [10]
-	EG	1.263 ± 0.008	0.839 ± 0.005	0.59	0.810 [12]
-	olive	1.175 ± 0.006	0.697 ± 0.003	0.43	0.621 [13]
